# Gambling in Sub-Saharan Africa: Traditional Forms and Emerging Technologies

**DOI:** 10.1007/s40429-022-00449-0

**Published:** 2022-10-25

**Authors:** Byron K. Y. Bitanihirwe, Tunde Adebisi, Christopher Bunn, Derrick Ssewanyana, Paul Darby, Paul Kitchin

**Affiliations:** 1 Humanitarian and Conflict Response Institute, University of Manchester, Ellen Wilkinson Building, Oxford Road, Manchester, M13 9PL UK; 2 School of Sport, Ulster University, Belfast, UK; 3 School of Social and Political Sciences, University of Glasgow, Glasgow, UK; 4 Temerty Faculty of Medicine, University of Toronto, Toronto, Canada

**Keywords:** Addiction, Africa, Gambling, Interventions, Policy, Technology

## Abstract

**Purpose of Review:**

The gambling industry in Africa has seen substantial growth and evolution over recent years with a growing body of literature describing these shifts. Here, we provide a narrative synthesis of the extant literature on the origins, trends and consequences of the expansion and intensification of the commercial gambling industry in sub-Saharan Africa with a reference for future research on gambling as a growing public health concern.

**Recent Findings:**

The historical shift and permeation of gambling in sub-Saharan Africa is diverse with evidence of certain countries following a neo-colonial logic. Advances in technology have made gambling more accessible and created new markets in Africa. A key motive driving gambling on the continent is a lack of stable employment. While the intensification and growth of Africa’s gambling industry has brought economic benefits to some African investors and individuals, this has been accompanied by a range of gambling harms. Legislation and policies designed to better regulate the gambling industry and redress these harms are needed. In this context, a small number of services and campaigns designed to mitigate gambling harms demonstrate promise, but more research is needed in this area.

**Summary:**

The gambling industry in sub-Saharan Africa has undergone a dramatic transformation. While it is true that the growth of the African gambling industry has provided an additional revenue stream to governments, it is also necessary to acknowledge the concurrent rise in gambling addiction and the health-related and social harms that it elicits. As such, designing effective regulatory measures and policy interventions that can reduce the public health burden of gambling harms is vital. However, these interventions need to take in to account the significance of cultural differences that exist among countries on the continent.

## Introduction

Recent years have seen a dramatic shift in the way people gamble in Africa. Specifically, online gambling on the continent has been driven by increasing digitization, expanding use of mobile devices and high internet penetration, in addition to accessible and more diverse payment options. This shift has reflected the wider global popularity of online gambling [1] and has been facilitated by technological advancements across the continent [2•, 3–5]. These developments have combined to create a dynamic, complex and fast-moving gambling landscape [6••]. The effects of the COVID-19 pandemic have further spurred individuals to shift to online gambling modalities [7•].

The surge of gambling establishments (legal and illegal) in Africa highlights the profitability of the industry with providers that include international corporations. Notably, revenue from gambling related advertising has increased substantially, driven by gambling on mobile technology [8]. In 2021, the total revenue from mobile gaming in the three largest African gambling markets (i.e. South Africa, Kenya and Nigeria) was placed in the region of half a billion US$ from mobile betting activities alone, and this figure is expected to grow further [9].

While it is appreciated that the growth of the African gambling industry has provided extra job opportunities and additional tax revenues for governments, it is also necessary to acknowledge the negative effects of gambling on the health and wellbeing of individuals, families, communities and society [10••, 11, 12]. In Africa, the high level of poverty and un(der)employment, especially among youth, has increasingly seen those from this vulnerable stratum of society turning to gambling due to distorted perceptions or beliefs about the potential of gambling as a viable, consistent source of income [13–16]. Importantly, distorted perceptions related to gambling have been reported to predict the frequency of gambling [17] and represent strong predictors of gambling-related harms [18].

Only a handful of studies exist from the African continent focusing on gambling harm prevention and intervention. These have addressed the effectiveness of financial education [19], counselling services [20], cognitive behavioural therapy [21, 22], motivational interviewing [23••] and awareness campaigns [24]. In this context, the recently launched Gambling Realities Africa Platform serves as a collective of researchers and practitioners which brings critical, evidence-based research to discussions and action to understand gambling and to reduce gambling-related harms across the African continent [25]. Specifically, this platform aims to create a space in which researchers, practitioners, policymakers and experts by experience can exchange ideas, foster collaborations and collectively pursue social change.

Although there has been a steady increase in the number of peer-reviewed publications relating to gambling in Africa, there remains a dearth of literature focusing on the development of the gambling industry across the African continent. As such, the purpose of this narrative review lies in providing insight into the question of historical shifts linked to gambling in sub-Saharan Africa (SSA) and aspects that spur gambling on the continent, and subsequent related harm. The paper is structured as follows. First, we outline the traditional forms and emerging technologies shaping gambling in SSA. Second, we describe the prevalence rates and demographics of gambling on the continent. Third, we discuss the underlying reasons driving gambling among Africans. Fourth, we touch on public health concerns related to gambling. We conclude by reflecting on the implications of gambling and policy recommendations that may concentrate on this area.

## Methods

We adopted the narrative review process [26] [27] to retrieve and analyze publications and literature on the origins, trends and consequences of the expansion and intensification of the commercial gambling industry in SSA. As with the initial steps of a PRISMA systematic review, this narrative review was guided by our purpose — to identify the origins, trends and consequences of the expansion and intensification of the commercial gambling industry in SSA. The search strategy involved identifying relevant databases which included PubMed, Scopus, MEDLINE, Embase, Web of Science (Science Citation Index and Social Science Citation Index) and Psych Info. This strategy combined various keywords relating to gambling in Africa (e.g. “Africa”, “compulsive gambling”, “gambling”, “gambling addiction”, “gambling prevalence”, “intervention”, “policy”, “prevention”, “problem gambling”, “regulation” and “sub-Saharan Africa”) and included both subject and free-text searches. We screened the reference lists of included articles (viz., we applied a snowballing approach) for any additional articles of interest. Our review also included grey literature (e.g. reports, policy literature, blog posts, government documents, white papers, dissertations) by using advanced Google targeted website searching. Using these systematic approaches, our narrative review was underpinned by a rigorous approach to searching, but given its exploratory nature diverges from the PRISMA guidelines by adopting this more inclusive approach to article selection. Additionally, our review did not deploy systematic quality assessment, which is a further departure from the systematic review approach. However, this was an appropriate choice, as it enabled us to produce a broad overview of the extant literature.

## Narrative Review

### What Are The Traditional Forms and Emerging Technologies Shaping Gambling?

Traditional forms of gambling and betting pre-date the recent, digitally mediated, intensification of commercial gambling products across the continent of Africa. As with many subjects of research, accounts of pre-colonial gambling practices across Africa can be hard to come by, in part due to the suppressive and erasing nature of colonial cultural violence, and also due to the predominantly oral nature of African cultures in the pre-colonial period. However, a limited body of scholarship describing traditional forms of gambling and betting does exist [28–32].

Of this body of scholarship, Reefe’s survey of ‘Gambling in Traditional Africa’ is likely the most informative, though heavily reliant on colonial-era commentators. He identifies two widespread varieties of indigenous gaming. The first is games of ‘pitch-and-toss’. One prominent form, *Abbia*, is identified as a West African game, which was suppressed by German colonisers. *Abbia* involves the game master tossing multiple wood chips made from nut shells, decoratively carved with images such as animals, weapons and human figures, with gamblers making predictions on how they will fall (face up or face down) [32]. Reefe describes how *Abbia* was entangled in complex forms of social exchange, citing an example of how a forest lord of what is now Southwestern Cameroon used his success to accumulate ninety-seven wives through gambling. This forest lord subsequently ‘loaned’ some of his wives to bachelors to secure loyalty. Such exchanges were so common, he argues, that the very word *Abbia* carried the dual meaning of ‘gambling chip’ and ‘loan woman’ [32].

The second variety, *Mancala*, goes by a multitude of names across the continent, with accompanying variations in form and is the most widespread. Reefe uses the term *wari-solo*, but also acknowledges the umbrella term *Mancala*, which is used to describe the wider ‘family’ of games to which *wari-solo* belongs [32]. Other names include Bao/Bawo (Kenya, Tanzania/Malawi), Oware (West Africa) and Alemungula (Sudan and Ethiopia). *Mancala* games are conventionally played between two people on wooden board, or a ‘board’ improvised or carved into the ground, and involve moving a series of small beads, beans or stones between cups in a strategic way to capture an opponent’s pieces. Bets placed on the outcome of the game are sometimes high-stakes and include cattle and daughters (i.e. waiving bride price), although the game is also played socially, without stakes. As with *Abbia*, then, *Mancala* games have been and are woven into complex relationships of social exchange.

During the slave-trade, colonial and post-colonial eras, the variety of gambling offerings grew across the continent. The introduction of card games has been traced to the slave-trade era but became mainstream during the twentieth century [32]. Sports betting in the form of football pools, horse racing and greyhound racing were introduced during the colonial period as part of the wider process of supplanting indigenous cultural forms with those of the coloniser [33]. In the post-colonial era, lotteries emerged as popular among new states looking for revenue streams [34•].

Since the digital era, notably from the start of the twenty-first century, the gambling products available across Africa have both diversified and intensified. Early forms of internet-mediated sports betting were offered via proxies, who owned computers and had internet access, enabling them to place bets using international platforms [35]. With increasing access to mobile internet, smart phones and mobile money services across much of the continent, African markets have opened up, with multiple corporations offering sports betting and online casino products. These new platforms take advantage of familiar business models, offering roadside franchise arrangements commonly used by mobile network and mobile money providers, as well as offering services in more formal structures and online via both browser and app-based platforms [4].

With greater presence and geographical penetration, gambling companies have diversified their product offerings across the continent. While sports betting [2•, 4] and horse racing [36] have become popular, they have also been utilised as mechanisms through which to expose bettors to a range of casino-style electronic gaming offers. Such offers include algorithm-based games of chance resembling roulette, virtual sports and simulated racing [37•]. In Malawi, the leading sports betting provider also gained a licence for alternate, more frequent forms of lottery, which draws numbers four times a day, 5 days a week [38]. Such a lottery format in this context is unusual and utilises mixed media: road-side kiosks, app and browser-based betting, television and social media broadcasts.

In the course of a century, then, large parts of Africa have transitioned from traditional forms of gambling through to a full range of pre- and post-digital offerings, introduced by external interests.

### What Are the Key Trends and Patterns in Gambling?

Sub-Saharan Africa is made up of 49 countries, most of which have casinos and other gambling establishments. While gambling is regulated in a majority of African countries (41, 83.6%), it is prohibited in 7 (14.3%) countries (Burundi, Eritrea, Guinea Bissau, Mali, Mauritania, Somalia, Sudan)—where Islam is the dominant religion, and Islam prohibits gambling [39••, 40]. Yet, evidence shows that Muslim communities in religiously diverse countries like Ethiopia, Senegal and Nigeria engage in gambling activities despite their prohibition under Islamic law [2•, 41, 42]. There are several factors—which are discussed in a later section—responsible for this reality, but technology or easy access to gambling platforms has been identified as a major factor responsible for the prevalence of gambling in Senegal—a country where over 95% of the populace are Muslims [42]. Likewise, in Nigeria, where Muslims are about half of the entire population—with a concentration in the Northern region—gambling platforms, whether online or onsite, could easily diffuse from bordering regions where gambling is permitted. Moreover, Islamic law—otherwise known as the Sharia—is not always strictly adhered to in a religiously diverse state like Nigeria [43]. In addition, Muslim Clerics have pointed out the differences among punishable offenses, as well as the jurisdiction of the punishment. Unlike adultery, fornication and stealing, gambling is punished by ‘*Allah*’, and *Allah* alone. Only in cases where the activity leads to the cheating or harming of another individual is it punished by *Sharia law*.

Beyond these important nuances, the extant research illustrates prevalence rates and gambling patterns among specific demographics including young people, defined by the African Youth Charter as aged between 15 and 35, in three geographical regions (Eastern, Western and Southern Africa). We provide a brief overview of what the scholarship reveals. In this section, we provide a country-by-country analysis—where possible—in relation to prevalence rates and gambling patterns for that particular country. The studies described here are divided by geographical region (i.e. Eastern Africa, Western Africa and Southern Africa), and our definition of a young person is based on the African Youth Charter where young people are defined as people between the ages of 15 and 35.

### Eastern Africa

Studies were identified from Ethiopia, Kenya, Rwanda, Tanzania and Uganda (Table 1). A single study involved a cluster of data from the East African Community (Kenya, Uganda, Burundi, Tanzania, Rwanda and South Sudan). Only a subset of the studies identified (27.8%) were conducted with minors and young people (11–35 years of age) as the focus.

**Table 1:** Summary of gambling studies and gambling prevalence in Eastern Africa

Country	Study type	Measure	Sample characteristics	Gambling prevalence	Legal age to gamble
EAC	Media analysis [47•]	N/A	18 M — 16 to 40 years of age	N/A	18 to 25 years old
Ethiopia	Cross-sectional study [41]	DSM-IV-J	227 M/195F — 12 to 21 years of age	6.9% were probable pathological	18 years old
Ethiopia	Cross-sectional study [44]	N/A	6 individuals — no age specified	N/A	18 Years Old
Ethiopia	Cross-sectional study [91]	DSM-IV-J	162 M/82 F — 18 to 22 years of age	7.3% were probable pathological	18 years old
Kenya	Randomized control trial [23••]	Questionnaire	198 M/30F — 1st–4th year University students	N/A	18 years old
Kenya	Exploratory research design^a^ [45]	Questionnaire	228 M/115F — 18 years and over	N/A	18 years old
Kenya	Cross-sectional study [51]	Questionnaire	203 M/25F — 1st–4th year University students	69.3% were disordered gamblers	18 years old
Kenya	Cross-sectional study [52]	Questionnaire	369 Secondary school students	32.3% had gambled at least once	18 years old
Kenya	Interview study [85]	N/A	Individuals over 18 years of age	N/A	18 years old
Kenya	Cross-sectional study [92]	PPGM	134 M/58F — 18 years and over	79.8% were pathological gamblers	18 years old
Kenya	Cross-sectional study [93]	Questionnaire	254 M/135 F — 18 years and over	N/A	18 years old
Rwanda	Cross-sectional study [11]	PGSI	104 M — 16 years and older	44% reported problem gambling	18 years old
Tanzania	Cross-sectional study [46]	Questionnaire	198 M/61F — 11 to 25 years of age	N/A	18 years old
Tanzania	Cross-sectional study [49]	Questionnaire	84 M/16F — 18 to 56 years of age	N/A	18 years old
Tanzania	Cross-sectional study [94]	Questionnaire	141 M/7F — 18 to 35 years of age	N/A	18 years old
Uganda	Cross-sectional study [48]	SOGS-RA	251 M/146F — 15 to 24 years of age	91% had at least 1 gambling problem	18 years old
					73% had gambled at least once
Uganda	Cross-sectional study [50]	Questionnaire	401 M — 18 to 59 years of age	21.6% engaged in betting daily	18 years old
Uganda	Case study [53]	Questionnaire	200 M — 20 to 40 years of age	N/A	18 years old

*EAC* East African Community (Tanzania, Burundi, Kenya, Rwanda, South Sudan and Uganda), *M* males, *F* females, *PPGM* Problem and Pathological Gambling Measure, *DSM-IV-J* Diagnostic and Statistical Manual of Mental Health 4th Version Adapted for Juveniles, *PGSI* Problem Gambling Severity Index, *SOGS-RA* South Oaks Gambling Screen Revised for Adolescents, *N/A* not available

^a^Binary choice model and 2 stage least square regression analysis

A variety of gambling practices take place in Eastern Africa ranging from playing cards, dice games, pool, lottery, bingo, scratch cards, flipping coins and sports betting to more country-specific practices such as *Carambolla* [41, 44]. It was widely found that sports betting represent the most common form of gambling that bettors participate in throughout Eastern Africa [45, 46, 47•, 53]. Prevalence levels of gambling among individuals in East Africa who have gambled at least once in their lifetime ranged from 32.3% in Kenya [52] to as high as 73% in Uganda [48]. In the compendium of studies that examined gambling trends among both men and women in Eastern Africa, men exhibited higher propensities to gamble and were more likely to screen positively for a gambling disorder.

### Western Africa

The gambling studies identified from Western Africa came from two countries: Ghana and Nigeria (Table 2). Card games, poker, slot machines, pool, sports betting and casinos constitute the more popular forms of gambling in Western Africa. As with Eastern Africa, the most common type of gambling in Western Africa was betting on sports [2•, 13, 14, 35, 54–65]. A majority of the studies identified (61.5%) were concerned with the gambling practices of minors and youth (10–35 years of age). Prevalence levels of gambling in study participants (i.e. gambled on a daily basis) from West Africa ranged from 18.1% in Nigeria [58] to 31.3% in Ghana [61]. In all studies that examined the gambling behaviours of both genders, males were found to gamble more frequently than females and were more likely to be diagnosed with a gambling disorder.

**Table 2:** Summary of gambling studies and gambling prevalence in Western Africa

Country	Study type	Measure	Sample characteristics	Gambling prevalence	Legal age to gamble
Ghana	Cross-sectional study [59]	ATGS	504 M/266F — 14 to 21 years of age	N/A	18 years old
Ghana	Cross-sectional study [61]	Questionnaire	136 M/4F aged 18 years and older	31.3% gambled at least once a day	18 years old
Ghana	Cross-sectional study [63]	Questionnaire	361 students of colleges of education	N/A	18 years old
Ghana	Cross-sectional study [64]	Interviews	20 M aged 17 to 35 years of age	N/A	18 years old
Ghana	Cross-sectional study [86]	DSM-IV-J	526 M/575F — 10 to 19 years of age	34.3% reported problem gambling	18 years old
Ghana	Cross-sectional study [87]	Questionnaire	42 M aged 19 to 34 years of age	N/A	18 years old
Ghana	Case study [102]	Interviews	25 individuals aged between 13 and 33	N/A	18 years old
Nigeria	Cross-sectional study [2•]	N/A	21 M/9F — 15 to 29 years of age	N/A	18 years old
Nigeria	Cross-sectional study [13]	SOGS-RA	237 M — 16 to 19 years of age	N/A	18 years old
Nigeria	Cross-sectional study [14]	SOGS-RA	356 M/15F 15 to 19 years of age	N/A	18 years old
Nigeria	Cross-sectional study [35]	Questionnaire	300 M aged 18 years and older	31% gambled at least once a day	18 years old
Nigeria	Cross-sectional study [54]	SOGS	131 M/15F — 18 to 74 years of age	N/A	18 years old
Nigeria	Cross-sectional study [56]	SOGS-RA	238 M aged 15 to 19 years old	N/A	18 years old
Nigeria	Cross-sectional study [57]	PGSI	160 M/14F — 21 to 34 years of age	N/A	18 years old
Nigeria	Cross-sectional study [58]	ATGS-8	428 M/321F — 16 to 30 years of age	N/A	18 years old
Nigeria	Case–control study [60]	G-SAS and IGT	69 M/8F — 18 to 35 years of age	N/A	18 years old
Nigeria	Cross-sectional study [62]	PGSI	186 M aged 21 to 31 years old	N/A	18 years old
Nigeria	Between group design [65]	BFI	126 M/14F — 18 to 26 years of age	N/A	18 years old
Nigeria	Cross-sectional study [95]	ATGS-8	165 M/32F — 18 to 34 years of age	18.8% gambled at least once a day	18 years old
Nigeria	Cross-sectional study [96]	Questionnaire	507 M aged 10 to 18 years old	57.2% gambled at least once in their life	18 years old
Nigeria	Cross-sectional study [97]	ATGS	507 M aged 10 to 18 years old	N/A	18 years old
Nigeria	Cross-sectional study [98]	Questionnaire	278 M aged 16 to 34 years old	N/A	18 years old
Nigeria	Cross-sectional study [99]	GBS	185 M/112F — 18 to 40 years of age	N/A	18 years old
Nigeria	Cross-sectional study [100]	GBS	185 M/112F — 18 to 40 years of age	N/A	18 years old
Nigeria	Cross-sectional study [101]	PGBQ	308 M/292F — 18 to 25 years of age	N/A	18 years old
Nigeria	Cross-sectional study [111•]	SOGS, ICD-11	376 M/77F — 18 to 50 years of age	30.5% reported gambling disorder	18 years old
		DSM-5			

*M* males, *F* females, *PGSI* Problem Gambling Severity Index, *PGBQ* Prevalence of Gambling Behaviour Questionnaire, *SOGS-RA* South Oaks Gambling Screen Revised for Adolescents, *ATGS* Attitude Towards Gambling Scale, *BFI* Big Five Personality Inventory, *IGT* Iowa Gambling Task, *G-SAS* Gambling Symptoms Assessment Scale, *GBS* Gambling Behaviour Scale, *DSM-IV-J* Diagnostic and Statistical Manual of Mental Health Fourth Edition Adapted for Juveniles, *ICD-11* International Classifications of Disease 11th Revision, *DSM-V* Diagnostic Statistical Manual of Mental Disorders Fifth Revision, *N/A* not available

### Southern Africa

Information pertaining to gambling was available from Malawi and South Africa (Table 3). Gambling activities in Southern Africa are varied, ranging from cards, dice games, betting on horses, pool, lottery, bingo, scratch cards, playing on stock or commodities market, flipping coins and sports betting to more country-specific practices such as *Fahfee* (or *umchina*), a form of lottery believed to originate from the early Chinese community in South Africa [66]. Sports betting is a favourite form of gambling observed in Southern Africa [4, 37•, 38, 67, 68]. This reflects Bunn et al.’s observation that the spread of sports gambling to Southern African countries follows a neo-colonial logic whereby English sporting products like Premier League football are pushed to English-speaking markets [37•]. Only a subset of the studies identified (27.8%) were conducted with young people.

**Table 3:** Summary of gambling studies and gambling prevalence in Southern Africa

Country	Type of study	Measure	Sample characteristics	Gambling prevalence	Legal age to gamble
Malawi	Media analysis [4]	N/A	N/A	N/A	18 years old
Malawi	Interview study [37•]	N/A	10 M — aged 18 to 35 years old	N/A	18 years old
Malawi	Case report [38]	N/A	1 M — aged 16 years of age	N/A	18 years old
Malawi	Cross-sectional study [69]	SOGS	1347 M/995F — aged 15 to 29 years old	15.6% of people had ever gambled	18 years old
South Africa	Cross-sectional study [20]	N/A	298 M/182F — aged 18 to 72 years old	N/A	18 years old
South Africa	Cross-sectional study [22]	SDI-PD	78 M/50F — aged 32 to 56 years old	N/A	18 years old
PG-YBOCS					
South Africa	Focus group workshop [66]	N/A	63 individuals aged between 21 and 60	N/A	18 years old
South Africa	Cross-sectional study [67]	SOGS	250 M/16F — aged 18 to 81 years old	31.2% were probable pathological gamblers	18 years old
South Africa	Socio-economic analysis [68]	Questionnaire	204 M/196F — aged 18 to 25 years old	N/A	18 years old
South Africa	Cross-sectional study [70]	Questionnaire	150 M/150F — aged 18 to 81 years old	68% of people had ever gambled	18 years old
South Africa	Cross-sectional study [103]	SCI-PG and	32 M/60F — aged 19 to 72 years old	N/A	18 years old
PG-YBOCS					
South Africa	Cross-sectional study [104]	PGSI	1500 M/1500F — aged 18 and older	3% at high risk of problem gambling	18 years old
South Africa	Cross-sectional study [105]	PGSI	582 M/318F — aged 18 to 81 years old	28.3% at high risk of problem gambling	18 years old
South Africa	Cross-sectional study [106]	SCI-PD and	100 M/100F — aged 18 and older	N/A	18 years old
PG-YBOCS					
South Africa	Cross-sectional study [107]	GAS	69 M/65F — aged 18 to 32 years old	N/A	18 years old
South Africa	Case–control study [108]	CTQ-SF	38 M/24F — aged 18 and older	N/A	18 years old
South Africa	Item response theory analysis [109]	PGSI	1532 M/1468F — aged 18 and older	N/A	18 years old
South Africa	Taxometric analysis [110]	PGSI	619 individuals aged 18 years and older	N/A	18 years old

*M* males, *F* females, *PGSI* Problem Gambling Severity Index, *SOGS* South Oaks Gambling Screen, *SCI-PG* Structured Clinical Interview for Pathological Gambling, *PG-YBOCS* Yale-Brown Obsessive Compulsive Scale Adapted for Pathological Gambling, *GAS* Gambling Attitude Scales, *CTQ-SF* Child Trauma Questionnaire Short Form, *N/A* not available

Prevalence levels of gambling in study participants (i.e. having gambled at least once) ranged from 16% in Malawi [69] to 68% in South Africa [70]. In the studies that engaged with both genders, men were more prone to gamble than women and were more likely to screen positively for a gambling disorder.

### Multi-Site Study

Insights into the prevalence and demographics of gambling in Africa are also available from a multi-site research study that included undergraduate students aged 16–30 from 25 countries across the Americas, Asia and Africa—including Cameroon, Ivory Coast, Nigeria, Madagascar, Namibia, South Africa and Mauritius. This revealed that overall, 8% of the students engaged in frequent gambling (i.e. gambling more than once in the past week) [71]. Furthermore, frequent gambling was positively and significantly associated with engagement in physical fights during the past 12 months [71].

### What Are the Factors Driving Gambling Trends?

In Africa, as explored above, there exists a history of indigenous gambling cultures that were present long before the predominance of European gambling operators in the continent [31, 32, 72–74]. However, these gambling operators have been able to penetrate deep into the African continent and to create markets, leveraging a number of factors including unfulfilled expectations (e.g. ambitions for financial security) among an enormous un(der)employed youth population seeking economic liberation, who have a pre-existing local/indigenous gambling culture, have access to mobile smartphones, are digitally literate, reside in a weak macro-level regulatory environment and are passionate about sport [2•, 12, 16, 75, 76]. Taking advantage of the extensive influence of sporting activities in many African societies, and other conditions aforementioned, gambling operators have designed a series of sports gambling products, that they market as a ‘panacea’ to economic hardship, offering hope for a ‘better life’ [77••, 78]. This has contributed to ‘normalising’ the engagement in sports gambling across the continent [79, 80].

In the attempt to understand the drivers of participation among gamblers, a series of factors have been identified. Some of these include unemployment, economic hardship, the pursuit of enjoyment, passion for sports, peer group influence, parental gambling, commercial adverts, technology and weak macro-level regulatory regimes [2•, 55, 64, 81, 82]. The research exploring these issues allows us to identify three categories of drivers: (1) poverty—the absence of money, (2) pleasure—the love for the game and (3) proximity—an opportunity for gambling. The influence of these drivers on gambling behaviours exists despite the various social concerns and inherent downsides to gambling activities [15]. This has been attributed to resilience, a willingness to engage in risky behaviours and an ability to negotiate challenges on the part of those Africans who engage in gambling. While being resilient ensures participation in sports gambling despite recurring losses [35, 83], the capacity for navigating challenging experiences sustains their pursuit of new paths to maximize winning [2•]. Since gamblers are sold the hope of a better life, it is therefore expected that many economically disadvantaged individuals in the continent will embrace the activity as a means to escape their harsh economic situations.

While pecuniary benefits driven by unemployment, economic deprivation and poverty have been argued as being the most compelling drivers of gambling in Africa, the pursuit of pleasure or passion for sports and the opportunity for the game are relatively less important factors [83]. For instance, football fandom in SSA often entails adopting one or more European clubs [35], commonly from multiple leagues (e.g. English Premier League, German Bundesliga, Italian Serie A, Spanish La Liga). Such fandom frequently imports the rivalries and historical disputes that exist between these European clubs. Gambling operators exploit these cultural trends through marketing strategies such as that used by *bet9ja*, whose brand tagline is captioned ‘reward for passion’.

### What Are the Public Health Concerns Related to Gambling?

Globally, gambling is increasingly being positioned as a public health issue, with academics, health practitioners, policy makers and activists seeking to highlight the range of harms gambling can engender [12, 76, 84••]. Framing gambling as a potentially harmful product draws on a range of evidence, notably that much of the industry likely derives the majority of its profits from those who are at risk of being harmed by their gambling [12].

Literature on African countries that has taken a public health perspective on gambling has emerged and dialogued with this recent shift in framing. A common observation and concern raised in this literature is that many young people participate in gambling to the detriment of personal or household finances, with consequences for their diet, hygiene, relationships and education, all of which have knock-on impacts on health [37•, 45, 68, 85]. Further common findings note that significant proportions of young people display problematic gambling behaviours, which are sometimes framed as addictions [11, 35, 41, 48, 50, 51, 58, 61, 86], and that various forms of mental distress accompanied these behaviours [2•, 38, 45, 47•, 51, 87]. The most recent development in the literature has been to identify connections between gambling and suicidality, with papers from Malawi [38] and on East African Community countries [47•] documenting cases of suicide among gamblers.

The evidence that gambling is detrimental to public health in African contexts has often been linked to legislative and regulatory weakness [39••]. Multiple studies report that underage gambling is common, pointing to limited regulation and enforcement [38, 47•, 48, 55, 58, 59, 86, 88, 89]. This is of particular concern, given the gambling harms identified above and the potential for adolescents to have their psychosocial development disrupted by gambling problems and their common sequelae. The widespread practice of underage gambling has led many to call for legislative, regulatory and enforcement responses which respond to digital gambling and the rapid growth in physical outlets in communities [4, 35, 37•, 38, 50, 64, 75, 78, 87, 88, 90•].

Despite the present evidence and numerous calls for intervention embedded in the public health-oriented literature on gambling across Africa, very little research has been conducted on what might be effective and how this could be implemented. One study has evaluated the efficacy of motivational interviewing for treating gambling disorder among university students in Kenya, finding the intervention to elicit statistically significant reductions in gambling disorder symptoms, frequency of gambling and amount wagered per bet [23••]. While showing promise, this is the only intervention that has been examined in the academic literature. A key research priority is therefore to develop and evaluate a range of interventions for African countries that can address gambling harms in culturally specific and competent ways.

## Conclusions

Gambling on the African continent is increasingly researched, with growing attention paid to its economic and public health ramifications. While gambling and betting practices on the continent date back to the pre-colonial era, it is imperative to recognize the increasing diversity in gambling/betting products, the growing access through digital and physical betting infrastructure and the intensifying aggressive marketing by the gambling industry in Africa. Sports betting, horse racing, casino-style electronic gaming and other algorithm-based games of chance like virtual sports are common examples of activities in the ‘new era’ of gambling on the continent. The extensive influence of sporting activities in many African societies; the economic hardship; the technological advancement characterized by increased access to internet, smart phones and digital platforms and the proximity to gambling avenues, coupled with weak legislative and regulatory environment, are some notable drivers of gambling among Africans. Evidence from this review emphasizes the importance of developing a more systemic approach in planning and implementing responsive policies or interventions aimed at lessening potential harms resulting from problem gambling on the continent such as mental and behavioural health problems and individual and household financial constraint. A systemic approach may include but not be limited to multi-disciplinary research, multi-sectoral stakeholder collaboration, deeper understanding of the socio-cultural and political contexts within which gambling occurs, as well as examining historical transitions in forms and patterns of gambling/betting practices on the continent. Of particular note, this narrative synthesis points to a current dearth and thus timely need for investment in implementation and evaluation research so as to devise culturally appropriate policy options and interventions which can effectively tackle gambling harms and their ramifications across the continent.
